# “Play by play”: A dataset of handball and basketball game situations in a standardized space

**DOI:** 10.1016/j.dib.2024.111265

**Published:** 2024-12-27

**Authors:** Bruno Cabado, Bertha Guijarro-Berdiñas, Emilio J. Padrón

**Affiliations:** a Universidade da Coruña, CITIC Research Center, A Coruña 15071, Spain; b CINFO CONTENIDOS INFORMATIVOS PERSONALIZADOS SL, Ciudad de las TIC, A Coruña 15008, Spain

**Keywords:** Sports, Players, Ball, Position, Velocity, Normalized, Game situation

## Abstract

This paper presents a synthetic dataset of labeled game situations in recordings of federated handball and basketball matches played in Galicia, Spain. The dataset consists of synthetic data generated from real video frames, including 308,805 labeled handball frames and 56,578 labeled basketball frames extracted from 2105 handball and 383 basketball 5-s video clips.

Experts manually labeled the video clips based on the respective sports, while the individual frames were automatically labeled using computer vision and machine learning techniques. The dataset encompasses seven classes of game situations: left attack, left counterattack, left penalty, right attack, right counterattack, right penalty, and timeout. In basketball, the penalty class refers to the free throws attempted by players after they have been fouled by an opposing player.

Each frame in the dataset is assigned to one of these classes, considering the game situation and specific context. Importantly, the dataset does not contain actual video frames; instead, it provides a synthetic, normalized representation of each frame in JSON format. This tabular data includes player, referee, and ball positions on a normalized field, player and referee velocities, and key regions on the court. Positions of players, referees, and the ball were automatically inferred in each frame by an object detector, followed by a tracking step to detect object positions across frames and compute the velocity vectors. Finally, the obtained coordinates underwent normalization through a perspective transformation, ensuring that the data remained unaffected by variations in camera configurations across different arenas and camera setups. We refer to this standardized coordinate space as the 'unified space'.

The dataset holds significant potential for reuse in various domains related to sports analytics and machine learning research. It can serve as a valuable resource for researchers, coaches, and sports enthusiasts, contributing to improvements in player performance, game strategies, match retransmissions, and sports-related technologies.

Specifications Table

**Table:** 

Subject	Data Science / Applied Machine Learning
Specific subject area	Game situations in sports
Data format	Analysed, Filtered
Type of data	Table
Data collection	The final synthetic data were generated through the processing of video footage from local basketball and handball matches in the Galicia region of Spain. The videos were manually segmented into frames and label them. Subsequently, they were processed to extract the positions of the players and balls, the velocities of the players and the important regions of the field from each frame. The recordings used to generate this dataset were captured with AXIS-P3818-PVE panoramic cameras between November 2021 and May 2022.
Data source location	*Institution: CINFO CONTENIDOS INFORMATIVOS PERSONALIZADOS SL* *City/Town/Region: A Coruña* *Country: Spain*
Data accessibility	Repository name: Zenodo Data identification number: 10.5281/zenodo.12607661 Direct URL to data: 10.5281/zenodo.12607661
Related research article	Bruno Cabado, Bertha Guijarro-Berdiñas, Emilio J. Padrón, Real-time Analysis of Indoor Sports Game Situations through Deep Learning-based Classification, Submitted to Engineering Applications of Artificial Intelligence. Under Review.

## 1 Value of the Data

• The labeled handball and basketball dataset provides a resource for developing and evaluating machine learning algorithms and models related to sports analysis.
• Researchers in sports science, computer vision, and machine learning can benefit from this dataset to analyze game strategies and identify factors that contribute to success.
• The dataset can be reused to develop predictive models for sports betting, injury risk assessment, sports analytics, game strategy optimization, real-time match retransmissions and other sports-related applications.

## 2 Data Description

**Fig. 1:**
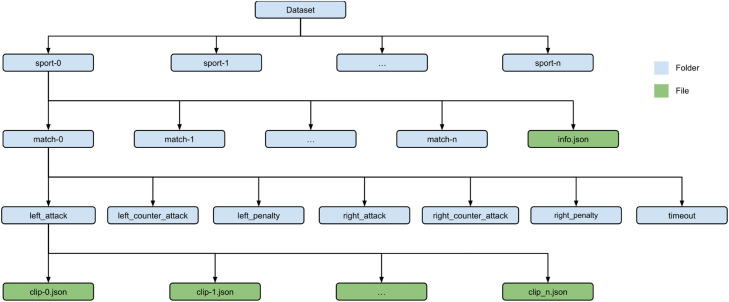
Folder structure of the provided dataset. The data is partitioned into several folders for the different sports. The ellipses (“...”) represent the possibility of an indefinite number of elements of a certain type (sports, matches and clips) that can extend from 0 to “*n*”.

This article describes the dataset presented in the linked repository [1]. The dataset is organized, as shown in Fig. 1, in a hierarchical file structure with a root directory containing subdirectories for each sport (currently, handball and basketball). This structure accommodates future expansion to include additional sports. Within each sport directory, there are subdirectories for each match. Each match directory contains subdirectories for the seven different classes of game situations (left attack, left counterattack, left penalty, right attack, right counterattack, right penalty, and timeout).

Each sport directory also includes an “info.json” file which specifies the matches that are used as the test set. Each class folder contains JSON files, with each file corresponding to a video clip labeled with that class. These JSON files provide the relevant data for each frame in the video clip. The hierarchical file structure provides an organized and efficient way to access and analyze the labeled data.

**Fig. 2:**
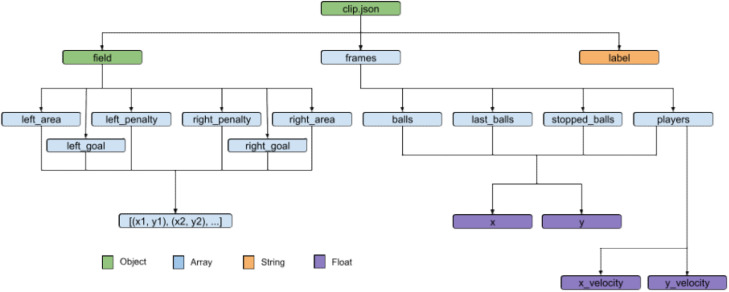
JSON structure of the provided clips in the dataset. Each clip has information about the key field regions, description of frames in the clip and the assigned class label.

Each “clip-x.json” file, as described in Fig. 2, contains data for all frames obtained from a single clip of a sport match, describing the positions of the players and balls in unified space and the velocity vectors of the players on the field at a particular moment in time. The data is stored in JSON format, following a schema that includes information about key regions on the playing field, the frames of the clip, and a label for the current game state (same as the parent folder name).

Specifically, the field object within the JSON file includes information about the left and right free-throw areas, the goals, and the 7-m marks for handball. In the case of basketball, the area regions represent the 3-point shooting zone and the 3-s zone. Each of these areas is represented by an array of two numbers, indicating the *x* and *y* coordinates of the points of the area.

The frame array contains information about the players and balls on the field at a given moment in time. Players include members of both teams and referees, as our dataset does not distinguish between them.

Each frame is an object with arrays for balls, last_balls, players, and stopped_balls. The balls array contains information about the position of all the detected balls on the field for that frame. The last_balls array includes the position of the last detected balls in a previous frame, useful for example if no ball is detected in some frames. The players array provides position and velocity data for each player on the field. The stopped_balls array contains information about any balls that have come to a stop on the field, indicating periods when the ball is not in active play. All positions on the court are expressed in a unified space, with width normalized between 0 and 2, and height between 0 and 1. This unified space was obtained by applying a four-point homography transformation separately to each side of the field on the original video frames.

Finally, the label field provides a string indicating the current state of the game. These classes were selected by sports broadcasting experts because they effectively represent handball and basketball games. Table 1 provides detailed information on these classes.

**Table 1:** List of the game situations considered in the dataset with their descriptions and number of occurrences.

Situation	Description	Occurrences	
		Handball	Basketball
Left Attack	Offensive move executed by the attacking team on the left side of the court.	111,772	17,227
Left Counterattack	Rapid offensive move launched by the attacking team on the left side of the court.	9615	1634
Left Penalty	Penalty or free shot executed on the left side of the court.	8852	2820
Right Attack	Offensive move executed by the attacking team on the right side of the court.	115,383	19,455
Right Counterattack	Rapid offensive move launched by the attacking team on the right side of the court.	9939	890
Right Penalty	Penalty or free shot executed on the right side of the court.	8549	5494
Timeout	Teams are in a game stop.	44,698	9058

**Fig. 3:**
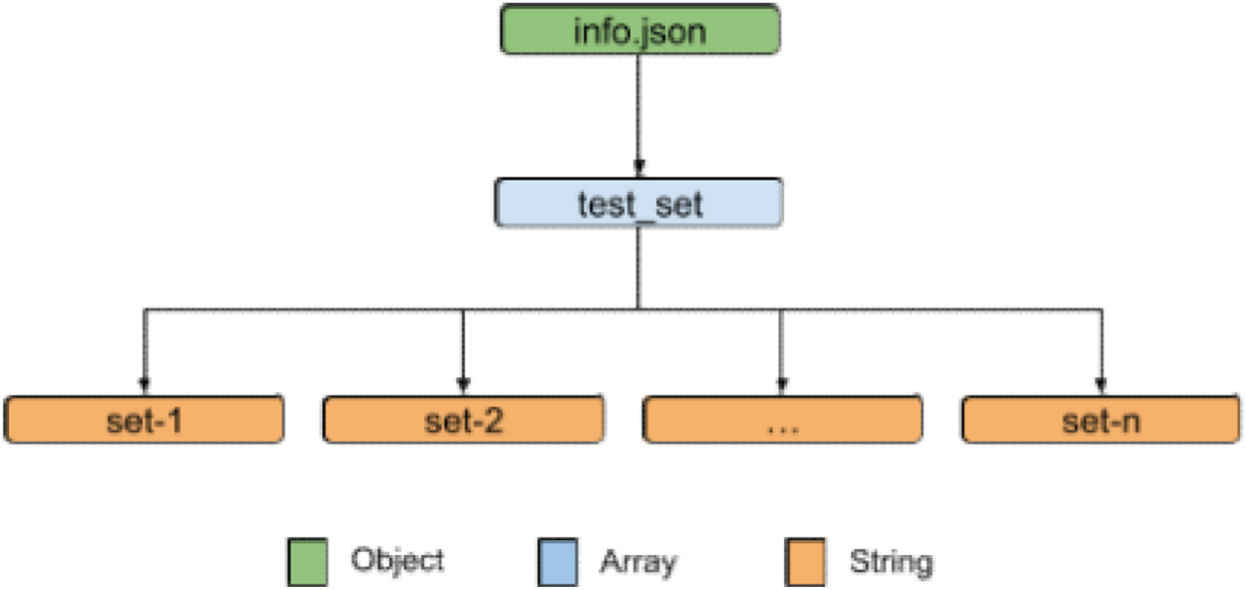
JSON structure of the provided info files in the dataset. This file describes the test set for each sport.

The “info.json”, as depicted in Fig. 3, is a JSON object with one key called “test_set”. The value of this key is an array of strings representing different test sets. Each string in the array refers to a specific match.

**Fig. 4:**
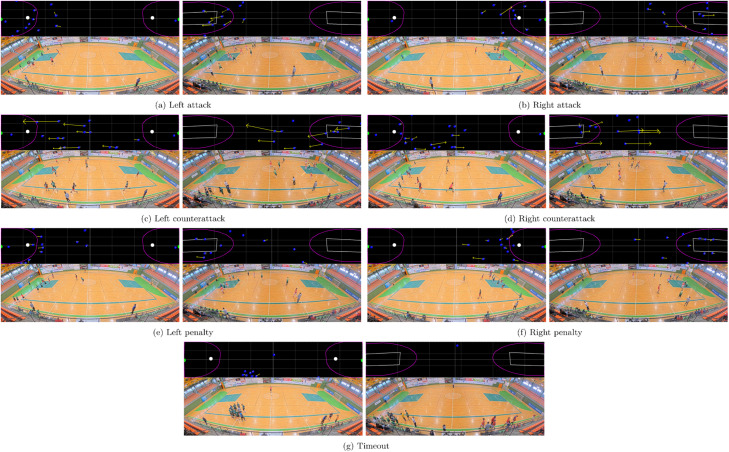
Examples of the different classes in the dataset for handball (left images) and basketball (right images). At the top of each image, the positions and velocities of players, the balls, and marked regions on the unified space.

Fig. 4 showcases examples of the available classes in the dataset for both handball and basketball. These images illustrate the diversity of game situations captured in the dataset, providing a comprehensive view of the different scenarios. Each image shows the positions and velocities of the players, the location of the ball, and key marked regions on the court, all represented in unified space. Below each unified space visualization, the corresponding video frame is displayed.

## 3 Experimental Design, Materials and Methods

### 3.1 Raw data obtention

The dataset was collected by recording videos of handball and basketball matches between November 2021 and May 2022 (for handball) and February and March 2022 (for basketball). The recordings were made using panoramic cameras of high resolution, 5120 × 2560 specifically. The matches were played by teams of diverse levels, genders, and ages. Only one camera was used for each location.

### 3.2 Data labeling

To label the different situations in the recorded videos, we used the tool **mpv-copy-time**, which allowed us to manually set the times when a situation occurred. Specifically, we paused the video at the beginning of each situation, marked the timestamps, and repeated the process for each situation. This resulted in a timestamp of the start time for each situation in the video.

### 3.3 Data cutting

Data cutting was performed on each situation obtained in the previous step of data labeling. To automate this process, we developed a script using **ffmpeg**, which takes the start time value for each situation. The script performs an initial fast search to 10 s before the start time, followed by a slow and precise search to start cutting at the exact start time point. We then cut 5 s of duration to obtain the desired situation. To improve the speed of the process, we used the HEVC codec running on GPU with the HQ preset and a 25 M bitrate.

### 3.4 Filtered data extraction

To extract the filtered data, we utilized machine learning and computer vision techniques on the videos obtained during the initial data cutting phase. Our system extracted crucial information from each video segment, including the positions of key field zones (e.g., areas, goals), player positions and velocity vectors (based on previous frames), and the ball's position. Additionally, we employed a four-point transformation to normalize the data to a 2D space, effectively mitigating camera distortion-related issues. The resulting information was saved for each frame in a JSON file, with one file for each labeled situation. This allowed us to obtain a detailed and structured dataset for analysis and modeling purposes.

To the authors' knowledge, although there are numerous sport-centered datasets, there are none that are labeled for the purpose of classifying game situations except for the one provided in Mures et al. [2], but it does not contemplate exactly the same classes and is limited to the sport of handball.

## Limitations

This dataset focuses exclusively on the game dynamics on the court, capturing the patterns of positions and movements of game agents. However, it does not include identifiers for individual players, does not differentiate between players from opposing teams, and does not explicitly distinguish between players and referees. Additionally, contextual information such as the game score, elapsed time, or other situational factors is not recorded. These limitations may restrict certain types of analyses that rely on agent-specific information, team-based strategies, or contextual insights related to game state and progression.

Finally, it should be noted that due to the nature of indoor sports games (handball and basketball), there is a clear class imbalance. Certain game situations such as regular plays and attacks are much more common than specific events like free throws or penalties.

## Ethics Statement

This dataset was collected in accordance with ethical principles and guidelines. The dataset was collected with the consent of the players, coaches and referees involved in the matches. The dataset does not include any personally identifiable information of humans involved in the matches. The data are anonymized adequately so that participants cannot be identified, and informed.

## CRediT authorship contribution statement

**Bruno Cabado:** Conceptualization, Methodology, Software, Validation, Formal analysis, Investigation, Resources, Data curation, Visualization. **Bertha Guijarro-Berdiñas:** Conceptualization, Methodology, Validation, Formal analysis, Investigation, Resources, Supervision. **Emilio J. Padrón:** Conceptualization, Methodology, Validation, Formal analysis, Investigation, Resources, Supervision.

## Data Availability

Zenodo"Play by Play": a dataset of handball and basketball game situations in a standardized space (Original data).

## References

[1] Cabado B., Guijarro-Berdiñas B., Padrón E.J. (2023). ``Play by play'': a dataset of handball and basketball game situations in a standardized space dataset. Zenodo.

[2] Mures O.A., Taibo J., Padrón E.J., Guitián J.A.I. (2024). A comprehensive handball dynamics dataset for game situation classification. Data Brief.

